# Perspective: The Promise of Virtual Reality as an Immersive Therapeutic

**DOI:** 10.1089/jmxr.2023.0003

**Published:** 2024-01-25

**Authors:** Todd Maddox, Charisse Y. Sparks, Liesl Oldstone, Michael Chibbaro, Josh Sackman, Emily Judge, Roselani Maddox, Robert Bonakdar, Beth D. Darnall

**Affiliations:** 1 AppliedVR, Inc., Van Nuys, California, USA.; 2 Inspire Medical Systems, Inc., Golden Valley, Minnesota, USA.; 3 Scripps Center for Integrative Medicine, Scripps Clinic, La Jolla, California, USA.; 4 Department of Anesthesiology, Perioperative and Pain Medicine, Stanford University School of Medicine, Palo Alto, California, USA.

**Keywords:** virtual reality, immersive therapeutic, neuroscience, chronic pain, cognitive behavior therapy

## Abstract

In this perspective, we discuss the promise of virtual reality (VR) as an immersive therapeutic (ITx) delivery device. We review the neurobiology of learning and show that VR broadly engages multiple learning systems in the brain in synchrony thus having the unique potential to increase the effectiveness and speed of therapeutic change. We examine one application of VR in the delivery of chronic pain management therapy. We discuss the strengths and weaknesses of current therapies such as pain education and cognitive behavior therapy and suggest that VR has the potential to deliver self-administered, accessible, low risk, in-home chronic pain therapy. We briefly review effectiveness data from an FDA-authorized 8-week self-administered behavioral skills VR program for chronic low-back pain called RelieVRx^®^, discussing the strengths and weaknesses of the data. We conclude by predicting that ITxs will continue to develop as our knowledge of their effectiveness in treating chronic pain and other mental and physical health conditions grows.

## Introduction

In this perspective, we discuss the promise of virtual reality (VR) as an immersive therapeutic (ITx) delivery device. We note that the goal of therapy is to change psychological and physical responses to environmental conditions, thus, to unlearn unhelpful cognitive and physiological patterns and to replace them with adaptive patterns. Learning and unlearning, and by extension therapy, involve structural and functional changes in the brain. We review the neurobiology of learning and show that VR can deliver content that broadly engages multiple learning systems in the brain in synchrony, thus having the unique potential to increase the effectiveness and speed of therapeutic change.

We keep the review high level thus making it approachable for non-neuroscientist clinicians and other health care professionals. Next, we examine an application of VR in the delivery of chronic pain management therapy. We discuss the strengths and weaknesses of current therapies such as pain education and cognitive behavior therapy (CBT) and suggest that VR has the potential to deliver self-administered, accessible, low risk, in-home chronic pain therapy.

We briefly review research testing RelieVRx^®^, an FDA-authorized 8-week self-administered behavioral skills VR program for chronic low-back pain (cLBP), noting the strengths and weaknesses of the data. This provides an example of the promise of VR for therapy and leads us to predict that ITxs will continue to develop as our knowledge of their effectiveness in addressing mental and physical health conditions (including chronic pain) grows.

## VR to Deliver ITxs

VR is being used to deliver therapeutic interventions to improve treatment outcomes for a range of mental disorders.^1^ Studies have shown the effectiveness of VR-delivered therapies in reducing fear of heights,^2,3^ social anxiety symptoms in individuals with autism spectrum disorder,^4^ symptoms of post-traumatic stress disorder in combat veterans,^5–7^ and reducing symptoms of depression in patients with major depressive disorder.^8–11^ VR has also been used in reducing drug craving and drug use behavior in individuals with cocaine use disorder.^12–15^

VR has emerged as a promising tool for delivering cognitive and physical rehabilitation, as well as chronic pain management therapies. It has been used to improve motor function, balance, gait, and other physical impairments in individuals with stroke,^16–20^ Parkinson's disease,^21–24^ and traumatic brain injury.^25^ VR-driven interventions have been shown to be effective in improving cognitive function in individuals with various neurological conditions,^26–29^ as well as in pain management for patients with chronic pain.^30–33^ Although these applications are relatively new and show promise, much more work is needed to validate their effectiveness across a broad range of patient populations.

Each of these examples involves unlearning unhelpful cognitive and physiological patterns and replacing them with adaptive patterns. As we outlined briefly in the next section, VR, unlike your traditional 2D mobile device, offers a powerful tool for unlearning and learning because it broadly engages multiple learning centers in the brain in synchrony.^34^

## Neurobiology of Learning and VR

In this section, we review the neurobiology of learning and how it relates to VR as a potential ITx delivery device. We keep the review high level, thus making it approachable for non-neuroscientist clinicians and other health care professionals, while fully acknowledging that this oversimplifies the breadth of knowledge and important nuance. [The interested reader is directed to several scientific outlets, such as peer-reviewed scientific journals such as “The Neurobiology of Learning and Memory” that examine the neurobiological mechanisms underlying learning and memory at all levels of analysis, ranging from molecular biology to synaptic and neural plasticity and behavior.]

“Learning is an experience. Everything else is just information.”

Albert Einstein

**FIG. 1.**
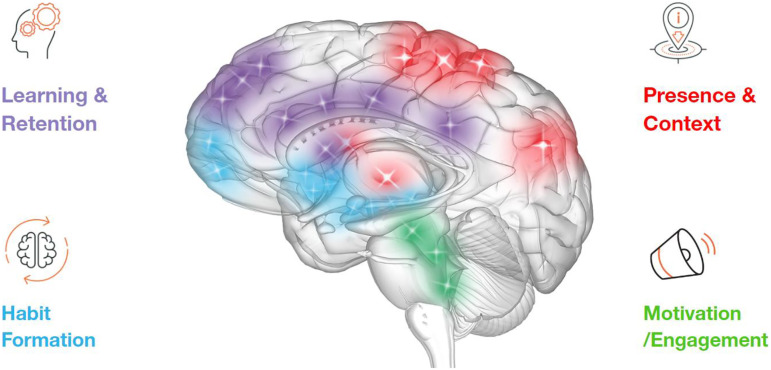
Schematic representation of learning systems in the human brain.

This learning-scientific quote from Albert Einstein is at the heart of why VR is effective for unlearning and learning and by extension has great potential as an ITx. The human brain includes four distinct learning systems^35–41^ displayed graphically in Figure 1.

As echoed by Einstein, experience is at the heart of learning. The *experiential learning system* in the brain represents the sensory and perceptual aspects of each unique learning context, whether visual (occipital lobes), auditory (temporal lobes), tactile or olfactory (parietal lobes). This system provides the rich foundation and scaffolding upon which unhelpful cognitive and physiological patterns are unlearned and adaptive patterns are learned. The *cognitive learning system* processes and stores knowledge and facts using working memory and attention.^38^ This system encompasses the prefrontal cortex and hippocampus. Processing in this system is adversely affected by anxiety, stress, and pressure.^42^

The *behavioral learning system* in the brain learns motor skills that, with repetition, can become automatic. The critical brain structure is the striatum that builds connections between environmental contexts (through the experiential and emotional learning systems) and motor behaviors.^38^ These striatal connections strengthen (learn) or weaken (unlearn) as a function of real-time reward and punishment feedback that mediates dopamine release. The *emotional learning system* in the brain provides rich motivational and emotional context to the environmental context represented in the experiential learning system. The critical brain regions are the amygdala and other limbic structures.^36,37^

Although these four learning systems in the brain are distinct, they are interconnected. In general, learning is optimized when all four learning systems in the brain are activated in synchrony, especially with the experiential learning system. The promise of VR, relative to other content delivery devices such as a 2D mobile device, is in its ability to engage all four learning systems in synchrony.^34^ When one dons a VR headset, he or she is transported into a new reality. This sense of “presence” follows from the strong engagement of experiential learning centers. This can reduce or eliminate the perceived distinction between the virtual and real world.

Information and knowledge can be conveyed through the cognitive learning centers, and through repetition, easily incorporated into VR programs, maladaptive automatic responses can be retrained toward adaptive symptom reduction. This change occurs within the context of an engaged motivational state created in VR.

## VR, Therapy, and the Brain: Chronic Pain Management

Here we briefly discuss one successful use case for VR as a therapeutic delivery mechanism, specifically, in the domain of cLBP. cLBP is the most prevalent form of chronic pain worldwide.^43^ A systematic review of the literature showed that no single brain area processes chronic pain, including cLBP.^44,45^ Rather, chronic pain is represented by a network of connected brain regions in the cerebral cortex.

**FIG. 2.**
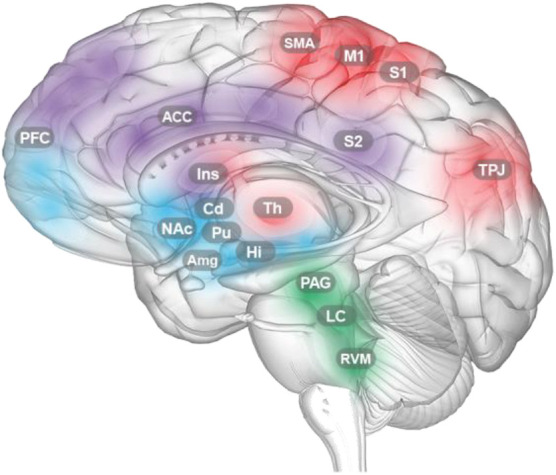
Neurobiology of pain. Schematic representation of neural networks involved in pain processing includes sensory/motor/multisensory regions (in red), pain affect/cognitive control regions (in purple), emotion/behavior regions (in blue), and descending modulation regions (in green). ACC, anterior cingulate; Amg, amygdala; Cd, caudate; Hi, hippocampus; Ins, insular cortex; LC, locus coeruleus; M1, primary motor cortex; NAc, nucleus accumbens; PAG, periacqueductal gray; PFC, prefrontal cortex; Pu, putamen; RVM, rostral ventral medulla; S1, primary somatosensory cortex; S2, secondary somatosensory cortex; SMA, supplementary motor cortex; Th, thalamus; TPJ, temporal–parietal junction.

As Figure 2 shows, pain processing can be represented by four neural networks: (1) sensory/motor/multisensory (in red; e.g., somatosensory and motor cortices), (2) pain affect/cognitive control (in purple, e.g., prefrontal cortex, anterior cingulate), (3) emotion/behavior (in blue; e.g., nucleus accumbens, putamen, anterior cingulate, insular cortex), and (4) descending modulation (in green; e.g., locus coeruleus, medulla). It is worth noting the extensive overlap between these neural networks associated with chronic pain (Fig. 2) and those associated with learning (Fig. 1).

As health care professionals reduce opioid prescribing for chronic pain treatment, alternative analgesic options are needed, particularly those that are self-administered, accessible, and low risk. Owing to substantial efficacy data, pain education and multisession therapist-led CBT are recommended as first-line treatments for cLBP.^46–49^ A growing body of literature suggests that the mechanism of action of CBT may be structural and functional changes in the brain's pain processing network (i.e., learning and unlearning^50–53^).

**FIG. 3.**
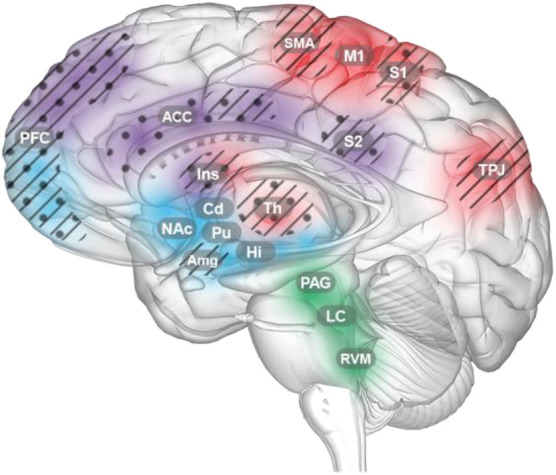
Overlap in brain region activation between pain processing centers (highlighted in red, purple, blue, and green), those regions affected by CBT (denoted by parallel lines), and those regions affected by VR-delivered pain distraction (denoted by solid black dots).

Interestingly, and as shown in Figure 3, many of the brain regions affected by CBT (solid parallel lines) overlap with the brain regions associated with chronic pain experience (colored), including dorsolateral prefrontal cortex, orbitofrontal cortex, ventrolateral prefrontal cortex, posterior cingulate cortex, and amygdala.

Despite the efficacy of multisession CBT, access to care remains poor due to barriers such as few trained therapists, health insurance limits, burdens associated with travel, and the 16 h of treatment time.^54^ Because of the scope and impact of cLBP, there is an urgent need for effective, in-home, accessible, low-risk treatments that are acceptable to cLBP sufferers. Critically, these treatments need to deliver the breadth of therapeutic content that is foundational to CBT, while offering the content repetitions needed to build pain coping skills and habits in cLBP patients. This is where ITxs, such as VR, hold promise as accessible and scalable therapeutic delivery solutions.

Seminal functional magnetic resonance imaging research by Hoffman and colleagues suggests that the brain regions engaged during VR-delivered pain distraction^55,56^ overlap extensively with the brain regions implicated in chronic pain and the brain regions affected by CBT, including dorsolateral prefrontal cortex, orbitofrontal cortex, ventrolateral prefrontal cortex, posterior cingulate cortex, and amygdala. These are highlighted visually in Figure 3 using solid black dots. Moreover, recent study using functional near-infrared spectroscopy and electroencephalography yields similar findings.^57–60^ Because VR engages the same brain regions affected by CBT and implicated in chronic pain, VR might provide an accessible, scalable immersive delivery system for chronic pain therapy.

## A Behavioral Skills-Based VR Program (RelieVRx)

Given the evidence suggesting overlap between possible VR and CBT brain mechanisms, it follows that several VR programs address chronic pain.^32,33,61^ Although several VR-based chronic pain programs exist, we focus this brief review on the only VR program that has obtained FDA authorization for cLBP, RelieVRx. We briefly review the program and its clinical efficacy.

**FIG. 4.**
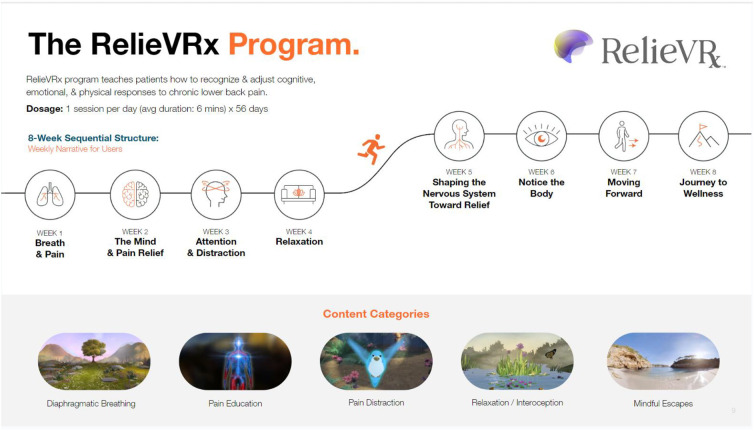
Overview of the RelieVRx^®^ program. RelieVRx utilizes an 8-week thematic, sequential structure with each week instantiating a specific theme. The FDA-authorized dosage is one experience per day with an average duration of 6 min. Each piece of VR content falls into one of five content categories.

RelieVRx delivers an 8-week self-administered behavioral skills-based VR program for cLBP. It is a multimodal program that integrates evidence-based skills such as diaphragmatic breathing, biofeedback, cognition and emotion regulation, mindfulness, and pain education into an 8-week therapeutic journey. The program includes daily immersive experiences (2–16 min in duration) organized into 8-week themes, with content falling into one of the five content categories: diaphragmatic breathing, pain education, pain distraction, relaxation/interoception, or mindful escape (Fig. 4). The program uses interactive biodata-enabled therapeutics that capture user exhalation through an embedded microphone to provide synchronized 3D visual and auditory biofeedback.

A double-blind, randomized, placebo-controlled trial compared RelieVRx with sham VR in *N* = 188 community-based adults with cLBP that was homogeneous (female: 76%, nonwhite: 8%, high school or less: 8%, without depressive symptoms) and clinically moderate (baseline pain intensity = 5.1; baseline pain interference = 4.8, disability = within normal range; sleep disturbance = mild).^62^ Clinically meaningful reductions (≥2 points^63^) in pain intensity (2.2) and pain interference (2.6) were observed at the end of treatment for the RelieVRx program that were significantly larger than for sham.

In total, 68% of RelieVRx participants^62^ achieved at least a 2-point reduction in pain intensity, pain interference, or both with an average reduction of 3.46 points. Pain reductions were durable to 24 months post-treatment although they were attenuated (pain intensity reduction = 1.2, pain interference reduction = 2.2^64^). Despite these promising findings, this trial was conducted during the height of the COVID-19 pandemic, in a homogeneous sample of participants leaving replicability and generalizability in question.

Maddox et al.^65^ conducted a double-blind, randomized, placebo-controlled trial comparing RelieVRx with sham VR in *N* = 1093 adults with cLBP that was demographically diverse (female: 72%, nonwhite: 32%, high school or less: 20%, depressive symptoms not excluded) and clinically severe (baseline pain intensity = 6.6; baseline pain interference = 6.2, disability = severe/completely disabled; sleep disturbance = moderate/severe).

Clinically meaningful reductions in pain intensity (2.0) and pain interference (2.3) were observed at the end of treatment for the RelieVRx program that were significantly larger than for sham.^63,66^ In total, 58% of RelieVRx participants achieved at least a 2-point reduction in pain intensity, pain interference, or both with an average reduction of 3.52 points.^67^ Durability tests of the RelieVRx program in this trial are forthcoming.

Although these initial results are promising, there is opportunity to fine tune the program as we better understand the patient characteristics associated with greater response and if the program is equally effective across age, gender, race/ethnicity, and socioeconomic status. For example, does the program work better for moderate or severe pain, is a dosage of one experience per day the most effective, and are certain content categories more effective than others and for whom?

Other promising ITxs for pain are entering the market. For example, Smileyscope (acute pain) and Cognifisense (chronic pain) both received FDA clearance to treat pain. Interestingly, both used RelieVRx as their predicate device. The biopsychosocial approach that underlies CBT and many successful chronic pain therapies is also foundational in treating some mental health conditions (for anxiety, depression, etc.).

Because content that captures the foundational principles of CBT and the biopsychosocial approach can be effectively delivered with ITx solutions, at scale, and with the repetitions needed to build coping skills and habits, we expect to see the number of ITx applications increasing and to include mental health conditions, as well as other forms of pain (chronic musculoskeletal pain, neuropathic pain, etc.).

## Pathway Toward Commercial Coverage

The U.S. Centers for Disease Control and Prevention (CDC) and U.S. Centers for Medicare and Medicaid Services (CMS)^68^ have made explicit calls for low-risk, accessible, effective, and durable nonpharmacological behavioral interventions for cLBP. VR-based therapies, such as RelieVRx, hold promise to answer these calls and ultimately to move toward broad commercial coverage. FDA authorization is an important step because it enables clinicians to prescribe the device to their patients.

Once a prescription is written, a device, such as RelieVRx, can be shipped directly to the patient's home where they can complete the therapy and ship the device back upon completion. Another important step toward coverage is the designation of a Healthcare Common Procedure Coding System (HCPCS) code for billing. RelieVRx has been granted a unique HCPCS code (E1905), assigned to the durable medical equipment benefit category and is on a path to CMS coverage. Establishing reimbursement and coverage criteria from CMS are key differentiators for any new technology. Without a benefit category assignment, any ITx will have difficulty gaining traction at scale with commercial payors and health plans that often look to CMS for guidance on payment methodology and eligibility.

## Summary and Conclusions

The goal of therapy is to change psychological and physical responses to environmental conditions, thus, to unlearn unhelpful cognitive and physiological patterns and to replace them with adaptive patterns. Learning and unlearning, and by extension therapy, involve structural and functional changes across multiple distinct neural networks. ITxs, such as VR, deliver content that broadly engage multiple learning systems in the brain in synchrony, thus having the unique potential to increase the effectiveness and speed of therapeutic change. We explore an application of VR in chronic pain management and compare it with the traditional therapeutic approaches.

We briefly describe RelieVRx, an FDA authorized 8-week self-administered behavioral skills-based VR program for cLBP, and two placebo-controlled randomized trials that demonstrate its effectiveness. Owing to its in-home accessibility, along with its ability to broadly engage multiple centers in the brain in synchrony, VR has the unique potential to deliver content that increases the effectiveness of therapeutic change through its direct effects on neural processing.

## Abbreviations Used

CBT: cognitive behavior therapy
cLBP: chronic low-back pain
CMS: U.S. Centers for Medicare and Medicaid Services
HCPCS: Healthcare Common Procedure Coding System
ITx: immersive therapeutic
VR: virtual reality
